# Sex and Gender in Myeloid and Lymphoblastic Leukemias and Multiple Myeloma: From Molecular Mechanisms to Clinical Outcomes

**DOI:** 10.3390/curroncol32040204

**Published:** 2025-03-31

**Authors:** Mohammad Amin Ansarian, Mahsa Fatahichegeni, Juan Ren, Xiaoning Wang

**Affiliations:** 1 Medical College, Xi’an Jiaotong University, Xi’an 710061, China; maansarian01@gmail.com (M.A.A.); mahsafatahi200@gmail.com (M.F.); 2 Department of Hematology, The First Affiliated Hospital of Xi’an Jiaotong University, Xi’an 710061, China

**Keywords:** sex differences, hematologic malignancies, treatment outcomes, gender disparities, pharmacogenomics

## Abstract

Biological sex and gender factors significantly influence the pathogenesis, progression, and treatment response in hematologic malignancies. This comprehensive review examines sex-specific differences in acute myeloid leukemia, acute lymphoblastic leukemia, chronic myeloid leukemia, and multiple myeloma through systematic analysis of the peer-reviewed literature published between 2014–2024 and identified through structured searches of PubMed, Web of Science, and MEDLINE databases. Epidemiological data demonstrate higher disease incidence (57% male vs. 43% female in MM, 63% male vs. 37% female in AML hospitalizations for ages 18–39) and inferior outcomes in male patients across malignancy types (5-year relative survival rates of 48.8% vs. 60.4% in females with AML), while female patients exhibit superior survival despite experiencing greater treatment-related toxicities. Our analysis reveals consistent sex-specific patterns in molecular mechanisms, including distinct mutational profiles, differences in immune system function, and sex-based pharmacokinetic variations that collectively suggest the necessity for sex-differentiated treatment approaches. The review identifies reproducible patterns across diseases, particularly in cytogenetic and molecular characteristics, with females demonstrating favorable prognostic mutations in leukemias and higher rates of chromosomal abnormalities in multiple myeloma. Despite these identifiable patterns, significant knowledge gaps persist regarding the underlying mechanisms of sex-based outcome differences. Incorporating sex and gender considerations into precision medicine frameworks represents a critical advancement toward optimizing treatment strategies and improving clinical outcomes for patients with hematologic malignancies.

## 1. Introduction

The goal of precision medicine in oncology is to focus on specific genetic profiles and molecular characteristics, especially for hematological malignancies [1,2], and tailor treatment accordingly. This approach has transformed patient care by improving diagnostic tools, improving prognosis, and selecting appropriate treatments [3]. Although precision medicine often focuses on molecular characteristics, there is growing recognition that incorporating sex and gender into personalized medicine is an important aspect. As highlighted by the European Society for Medical Oncology (ESMO) workshop on Gender Medicine and Oncology, integrating sex and gender dimensions into clinical research and practice is crucial for advancing personalized medicine approaches and addressing disparities in cancer outcomes [4].

Although the impact of biological sex on cancer pathogenesis is unknown, there is evidence that cancer is quite different in men and women because of differences in tumor biology, immune system function, body structure, drug use, etc. [5]. Male patients show consistently higher incidence rates across most hematologic malignancies, with a male predominance of 55–60% in AML, 57% in MM, and 60–63% in T-ALL [6]. The influence of these factors may have important implications in determining disease risk, symptoms, and response to treatment. Although the role of gender in cancer is becoming increasingly clear [7], there are still significant gaps in the research, especially in hematological malignancies. This paper aims to fill this gap by thoroughly reviewing the existing evidence on sex and gender differences in hematological malignancies and their impact on epidemiology, pathophysiology, clinical manifestations, disease progression, and response to treatment. We chose to focus specifically on leukemia subtypes and multiple myeloma due to their distinctive sex-specific patterns in incidence, molecular profiles, and treatment responses, which provide compelling models for understanding how sex and gender influence disease biology. Unlike lymphomas, which represent a highly heterogeneous group of over 80 distinct entities with variable sex distributions [8], leukemias and multiple myeloma offer more consistent sex-specific patterns that allow for meaningful comparison across disease subtypes.

## 2. Materials and Methods

In January 2024, we conducted a targeted literature search in PubMed, Web of Science, and MEDLINE databases. We searched for publications examining sex and gender differences in hematological malignancies, focusing specifically on leukemia and multiple myeloma. Search terms combined sex-related keywords (“sex”, “gender”, “male”, and “female”) with disease-specific terms and outcomes of interest (epidemiology, clinical manifestations, prognosis, and treatment outcomes).

Articles published between 2014 and 2024 were prioritized to ensure contemporary relevance, though seminal earlier studies were included where appropriate. Selection criteria focused on studies that specifically addressed sex-based differences in disease characteristics, molecular profiles, or treatment outcomes. A bibliographic review of selected papers identified additional relevant articles.

This review emphasizes evidence for biological sex differences while acknowledging the complex interplay with gender-related sociocultural factors that influence disease outcomes.

## 3. Defining Sex and Gender: A Crucial Distinction

Biological sex and gender are distinct but related concepts. Sex refers to the biological differences between men and women, including chromosomes, anatomy, physiology, and hormone profiles [9]. Although often treated as a binary, it is important to recognize the existence of intersex variations and the range of biological sex characteristics. Gender, on the other hand, is a sociocultural construct that encompasses the roles, behaviors, identities, and expectations associated with being male or female in a particular social context [10]. It is shaped by social norms, cultural values, and individual experiences, developing over time and varying across cultures.

Both sex and gender have a significant impact on health, disease development, and even environmental influences [11,12]. Gender influences physiological processes such as immune response and drug metabolism while also influencing exposure to pathogens, access to healthcare, and behaviors that affect health-promoting behaviors [11]. Understanding sex and gender differences, especially their interactions, is essential to advancing personalized medicine, reducing inequities in healthcare, and improving research designs in basic and clinical trials [13]. Unfortunately, the terms are often used interchangeably, creating scientific and legal confusion that can impede progress and harm patients [9].

Separating gender-specific biological influences from gender-related socio-cultural influences is complex, as these factors interact to affect health outcomes [14]. Research has highlighted the importance of considering both biological and sociocultural sex to understand health disparities [15]. Oversimplifying gender differences can lead to misinterpretation of data and reinforce stereotypes [16]. Hormones play a key role in these interactions, but their effects are more complex than often portrayed [17]. To move the field forward, researchers must embrace a critical approach that recognizes the complexity of gender interactions and avoids binary thinking [16].

### Addressing the Challenges in Data Collection and Reporting

A major challenge in research on sex and gender is inconsistency and inadequacies in data collection and reporting. Many studies do not adequately collect and report data stratified by sex and gender, preventing meaningful conclusions from being drawn about their respective contributions to health outcomes. Even when sex is specified, it is often treated as a binary variable, ignoring intersex individuals and the range of assigned gender at birth characteristics. Gender is often confounded with sex, obscuring the complex interplay of biological and sociocultural factors.

Standardized definitions and reporting practices are essential to address these challenges. Clear and consistent definitions of sex and gender are essential to ensure that research findings are comparable and generalizable. Reporting guidelines should be introduced to encourage researchers to take measures and collect and report data stratified by sex and gender, recognizing the limitations of binary classifications. This includes showing how sex and gender were assessed (e.g., self-report, biological measurements, etc.) and providing detailed demographic information. Researchers should be encouraged to explore the full range of gender identities and experiences beyond simple comparisons of “male” and “female”. We recognize that this review’s use of binary sex categorizations is a limitation that reflects larger issues in the field, even if it is mostly the result of present limitations in research technique and available data. In order to fully capture the range of biological sex traits and gender identity in hematologic malignancy outcomes, future research should strive toward more sophisticated methodologies.

## 4. Biological Sex and Hematologic Malignancies: Mechanisms and Manifestations

### 4.1. Genetic and Hormonal Influences

Sex chromosomes, sex hormones, and genetic factors have a significant impact on the development and progression of hematopoietic malignancies. Loss of sex chromosomes, especially tumor suppressor genes in the pseudo-autosomal region of the X chromosome, is frequently observed in these diseases [18]. For example, studies have shown that X chromosome loss in older men is associated with an increased risk of bone marrow malignancies [19].

Sex hormones play an important role in both normal and malignant hematopoiesis. Men are at higher risk for most hematologic malignancies, which may be because of differences in sex hormone levels [20]. This is demonstrated by the expression of functional estrogen and androgen receptors on hematopoietic stem cells, suggesting a direct hormonal regulation of blood cell development [21].

Genetic predisposition to hematopoietic malignancies is associated with various inherited mutations [22], like the X-linked GATA1 gene, which is essential for proper development of erythrocytes and megakaryocytes: it can harbor mutations that lead to diseases such as Diamond–Blackfan anemia and transient myeloproliferative disorder [23]. Another important example is the RUNX1 gene. Mutations in this gene interfere with normal hematopoiesis and are common in several types of leukemia [24].

Biological differences between sexes also affect immune cell function and development [25]. These differences are reflected in the differential susceptibility of men and women to various hematological malignancies. For example, chronic lymphocytic leukemia occurs more frequently in men, which may be because of the combined influence of sex hormones and X-linked immunoregulatory genes [26].

### 4.2. Immune System Variations

Sex differences significantly impact immune function and susceptibility to hematologic malignancies. Females possess stronger immune responses due to the presence of two X chromosomes, with approximately 15% of genes on the inactive X chromosome escaping silencing. Key X-linked immune genes like TLR7, TLR8, CD40L, and KDM6A enhance immune surveillance in females, potentially contributing to their lower incidence of certain blood cancers. Males, conversely, show increased susceptibility to malignancies like acute myeloid leukemia (AML) and multiple myeloma (MM), with males representing 57% of MM cases and experiencing AML hospitalization rates significantly higher than females (33.8 vs. 23.3 per 10,000 in the 60–79 age group) [27]. This vulnerability is partly attributed to the loss of the Y chromosome (LOY) in immune cells, which occurs in approximately 1.6% of males at low levels. LOY affects various immune cell populations (27% in NK cells, 23% in monocytes, 7% in B lymphocytes, and 3% in T lymphocytes) and is associated with clonal expansion in hematopoietic stem and progenitor cells (HSPCs), promoting leukemogenesis [28]. These chromosomal differences, combined with hormonal factors (estrogen’s immunostimulatory effects versus testosterone’s immunosuppressive effects), create distinct immune microenvironments that influence hematologic malignancy development, progression, and treatment outcomes between sexes.

Sex-specific immune differences significantly impact cancer immunotherapy responses. Men typically show 20–30% better overall survival with immune checkpoint inhibitor monotherapy, while women demonstrate 15–25% improved outcomes with combination therapies. This pattern stems from women’s more robust immune systems (influenced by the ~118 miRNAs on the X chromosome compared to only four on the Y chromosome) and estrogen’s immunostimulatory effects, which can produce stronger anti-tumor activity when properly directed through combination approaches. Men’s responses to monotherapy may be partially explained by the 27% LOY observed in NK cells and 23% in monocytes, affecting approximately 500 immune-related genes [29]. These differences are relevant in hematological malignancies, such as acute myeloid leukemia (AML), where the immunological microenvironment significantly affects disease progression and treatment efficacy [30].

Understanding these sex-specific immune differences is crucial to advance personalized medicine approaches. The different immunological profiles between men and women suggest that sex-specific treatment strategies may be necessary to optimize treatment outcomes in hematological malignancies [31]. For example, considering sex-specific immune responses when developing immunotherapy protocols may improve treatment efficacy and patient outcomes.

### 4.3. Pharmacogenomics

Sex has a significant impact on drug metabolism, pharmacokinetics, and pharmacodynamics in the treatment of hematological malignancies, leading to important differences in treatment efficacy and toxicity between men and women. Female CML patients demonstrate 23% higher dose-normalized plasma imatinib concentrations than men (0.0043 vs. 0.0035 L^−1^), but they achieve higher MMR rates (80% vs. 45%, *p* = 0.018) [32,33] This is a clear pattern in hematological malignancies, where men experience higher mortality despite often receiving similar treatment protocols [34]. These differences are because of both genetic variation and the action of sex hormones, which affect how drugs are processed and used in the body [35].

Patients often respond differently to drugs, usually with increased exposure because of longer half-lives. This can lead to increased toxicity but sometimes may also contribute to improved survival outcomes [36]. For example, in the treatment of acute myeloid leukemia, sex-specific polymorphisms in drug-metabolizing enzymes have been shown to affect both the efficacy and cardiotoxicity of anthracycline-based treatment [37]. Despite the obvious importance of these sex differences, they are often overlooked in the design of clinical trials and treatment protocols [38]. To address these differences, researchers have proposed several approaches, including gender-tailored dosing strategies and enhanced therapeutic drug monitoring practices [39]. Understanding and addressing these sex differences in drug use is critical to developing more personalized and effective cancer treatments while minimizing side effects [40].

## 5. Sex Differences in Hematologic Malignancies: A Disease-Specific Review

Sex differences play a fundamental role in the development, course, and treatment outcomes of hematopoietic malignancies. In this section, we comprehensively analyze these differences in two major hematologic malignancies (leukemia and multiple myeloma) and consider the epidemiological patterns, clinical manifestations, prognostic factors, and treatment responses. By systematically analyzing sex-specific variations in each disease, we aim to uncover key differences that can lead to more personalized treatment approaches and improve treatment outcomes.

### 5.1. Leukemia

**Figure 1:**
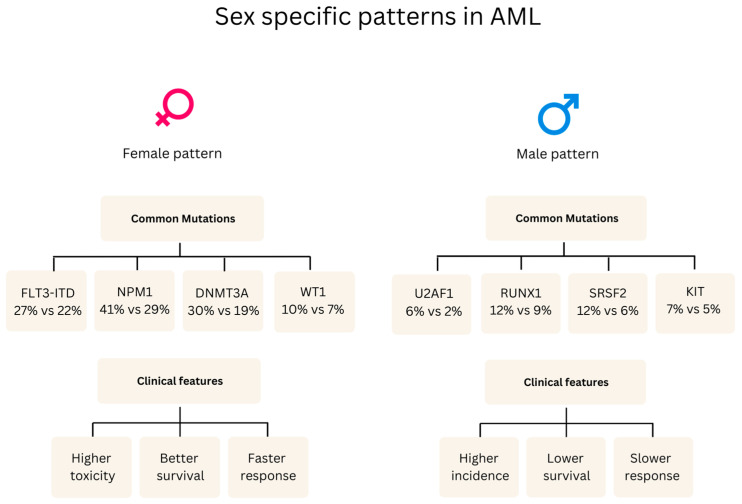
Sex-specific patterns in AML reveal distinct genetic and clinical profiles. Female patients commonly exhibit FLT3-ITD, NPM1, DNMT3A, and WT1 mutations, associated with higher treatment toxicity but better survival (51% vs. 42% 5-year OS, *p* = 0.005) and faster response rates (MMR 80% vs. 45%, *p* = 0.018). Male patients who show higher incidence (977 vs. 749 in the cohort) typically present with U2AF1, RUNX1, SRSF2, and KIT mutations, correlating with poorer outcomes and slower treatment response [49].

Significant sex differences exist in the major subtypes of leukemia. Logical patterns emerge in epidemiology, clinical presentation, disease biology, and treatment outcomes. Males consistently show 10–15% higher incidence rates across AML, ALL, and CML, alongside inferior survival outcomes (48.8% vs. 60.4% five-year relative survival in AML) [41]. In this section, we present a table showing a comprehensive analysis of biological sex characteristics in acute myeloid leukemia (AML), acute lymphoblastic leukemia (ALL), and chronic myeloid leukemia (CML). The important role of biological sex as a biological variable is highlighted in both research and clinical treatment (Table 1) (Figure 1).

**Table 1:** Sex-specific characteristics in major leukemia subtypes. This table presents a comprehensive analysis of sex differences in acute myeloid leukemia (AML), acute lymphoblastic leukemia (ALL), and chronic myeloid leukemia (CML) across four key domains: epidemiology, clinical manifestations, prognostic factors, and treatment response. Each cell contains key findings from the cited literature documenting sex-specific patterns in disease presentation, progression, and outcomes.

Category	AML	ALL	CML
Epidemiology	• AML hospitalizations peaked in the 60–79 age group (males: 33.8, females: 23.3 per 10,000) with a significantly higher rate in males aged 18–39 (23.6 vs. 7.7 per 10,000, *p* < 0.01) [27]. • Upward trend for 1990–2017 (from 63.84 × 10^3^ in 1990 to 119.57 × 10^3^ cases in 2017, increasing by 87.3%, EAPC = 0.56, 95% CI 0.49~0.62) [42].	• >90% survival in high-income countries [43]. • Boys with B-cell acute lymphoblastic leukemia (ALL) exhibited inferior 5-year event-free survival (84.6% vs. 86.0%, *p* = 0.009) and overall survival (91.3% vs. 92.5%, *p* = 0.02) compared to girls, primarily due to increased central nervous system relapses [44]. • B-cell (24 subtypes) and T-cell (10 subtypes) acute lymphoblastic leukemia (ALL) demonstrate a sex disparity, with T-ALL more common in males (63%) and B-ALL more common in females (58%) [45].	• Higher male incidence: Studies indicate a higher risk of CML in males, observed across age groups and supported by data from both general populations and specific cohorts like A-bomb survivors [46]. • Sex-specific transcript distribution: e13a2 (b2a2) is more frequent in males (39.2% vs. 36.2% in females); rare transcripts are more frequent in females (2.27% vs. 1.69% in males) [47]. • e14a2 (b3a2) and treatment response: Patients with e14a2 (b3a2) showed significantly better complete cytogenetic response at 12 months (78.6% vs. 21.4% for e13a2/b2a2) [48].
Clinical manifestations	• Males: ASXL1, SRSF2, U2AF1, RUNX1, and KIT mutations; aggressive features [49]. • Female-predominant mutations: FLT3-ITD, DNMT3A, NPM1, and WT1 [49].	• Males exhibit a higher prevalence of T-ALL (83%) and B-ALL (89%) and increased central nervous system involvement, particularly in B-ALL (22% of cases, mostly in males) compared to T-ALL (4% of cases) [50]. • Females show a higher frequency of early B-phenotype ALL associated with elevated cell counts [51], which contrasts with the general findings in [50] of a higher overall B-ALL prevalence in males (89%). • RASSF2 SNP (rs7704443): The minor allele (G) of this SNP was found to be significantly more frequent in males with childhood ALL, with an odds ratio (OR) of 1.7 (95% CI = 1.3–2.2) [52].	• Males: Higher WBC counts, larger spleen, earlier diagnosis [53]. • Females: Higher platelets, lower hemoglobin, later diagnosis [54]. • The disease predominately affected children who were older than 10 years (67% of the patients), with a higher prevalence in boys than girls (gender ratio: 1.5) [55].
Prognostic factors	• Inferior survival in males: Males with myeloid malignancies showed a lower 5-year relative survival rate of 48.8% (95% CI 46.5–51.2) compared to females, who had a rate of 60.4% (95% CI 57.7–62.9) [41]. • Black adolescent and young adult patients with AML, particularly those aged 18–29 years, experienced worse outcomes compared to White patients, including higher early death rates (16% vs. 3%), lower complete remission rates (66% vs. 83%), and decreased 5-year overall survival (22% vs. 51%). These disparities persisted even within specific cytogenetic groups [56].	• While historically, boys with ALL have experienced inferior survival, this study of pediatric B-ALL patients treated on Children’s Oncology Group trials between 2004 and 2014 did not find statistically significant differences in 5-year event-free survival (EFS: 85.3% for boys vs. 86.4% for girls, *p* = 0.07) or overall survival (OS: 89.8% for boys vs. 90.4% for girls, *p* = 0.17) between sexes [44].	• In low- and intermediate-risk CML patients (according to the Sokal score), females had a statistically significant higher cumulative incidence of major molecular response (MMR) at 12 months compared to males (70.3% vs. 52.9%, *p* = 0.037) [32]. • Females with CML in the chronic phase were more likely to achieve an early complete cytogenetic response (CCyR) at 3 months (HR 1.38 [95% CI 1.02–1.87], *p* = 0.03) and a major molecular response (MMR) at 6 months (HR 1.34 [95% CI 1.03–1.74], *p* = 0.03) compared to males. Females also had a higher cumulative incidence of MMR at 12 months (66.1% vs. 56.7%, *p* = 0.03) [57]. • Female sex has been identified as a predictor of durable deep molecular response, which is often a prerequisite for attempting treatment-free remission [57,58].
Treatment Response	Anthracycline cardiotoxicity varies by sex/life stage: Prepubertal females: Higher risk (RR 1.89, CI 1.28–2.78) Premenopausal females: Lower risk (16.5% vs. 7.3% hospitalization) Postmenopausal: No significant difference. Dexrazoxane protection is stronger in females (*p* = 0.019) [59].	• Clinical trials show females have better survival outcomes (42% vs. 16% of trials) but more toxicity than males (13 vs. 22 trials) [60]. • Sex-specific drug effects: - Vincristine increased non-void contractions by 700% in females compared to only 180% in males [61] - Asparaginase: males are more likely than females to develop severe hypertriglyceridemia, with 66% of grade 4 cases occurring in males compared to 34% in females [62]. • Female survivors showed significantly better overall metacognition (*p* = 0.024) compared to males following cisplatin/carboplatin treatment [63].	TKI therapy: Women have 23% higher dose-normalized plasma imatinib concentrations than men (0.0043 vs. 0.0035 L^−1^). Women achieve higher MMR rates (80% vs. 45%, *p* = 0.018). Women experience 12% more samples with high (>2mg/L^−1^) imatinib levels (34.9% vs. 22.6%) [32,33]. Age-related treatment adequacy: Elderly women receive 24% fewer doses (62 vs. 81 doses/quarter) than elderly men [64].

Biological sex is an important biological variable in all major leukemia subtypes. Interesting patterns emerge that significantly affect the course of the disease. Despite the differences in the underlying molecular mechanisms, men consistently have a higher incidence and worse survival in AML, ALL, and CML. In AML, males experience a 5-year relative survival rate of 48.8% compared to 60.4% in females (*p* < 0.05). In CML, females achieve higher major molecular response rates at 12 months (70.3% vs. 52.9% in males, *p* = 0.037). Remarkable patterns are seen in the mutational profiles. Female patients with AML and ALL have mutations associated with a better prognosis (FLT3-ITD, NPM1, DNMT3A in AML, and early B phenotype in ALL), while men have a higher frequency of high-risk mutations. Response to treatment presents an interesting paradox: women experience more treatment-related toxicity yet achieve better survival outcomes, which is clear in response to targeted therapy in ALL and CML. In CML, women experience greater treatment intolerance but better molecular responses and higher rates of treatment-free remission. These consistent sex-specific patterns in disease biology, disease progression, and treatment response highlight the need for sex-specific approaches in the treatment of leukemia, which remains largely underappreciated, with only 0.5% of oncology studies reporting sex-specific outcomes [60].

### 5.2. Multiple Myeloma (MM)

#### 5.2.1. Epidemiology

Multiple myeloma shows clear sex-specific patterns, with men accounting for 57% of cases across geographic regions and age groups [65]. Although the incidence is low, women have specific high-risk genetic features, including higher rates of IGH translocations, higher frequency of del(13q), and higher prevalence of +1q. In a study of 146 MM patients (88 males, 58 females), cytogenetic studies showed an abnormal-to-normal (A:N) ratio of 0.53 in females and 0.13 in males (*p* = 0.003244), while FISH studies showed an A:N ratio of 2.87 in females and 1.32 in males (*p* = 0.023312) [66]. In an analysis of 1960 MM patients, sex was associated with overall survival (OS) on univariate analysis (median OS 44.8 months female vs. 49.9 months male, *p* = 0.02) but was not significant in multivariate analysis. Similarly, an analysis of the Myeloma XI trial found no sex differences in progression-free survival (PFS) or OS. There were more males than females in both datasets: 55.5% males vs. 44.5% females in SEER (Surveillance, Epidemiology, and End Results) and 60.4% males vs. 39.6% females in MMRF (Multiple Myeloma Research Foundation) data. The impact of sex on survival is controversial, with some studies finding a worse prognosis in women [6] and others finding no significant difference [65,67]. Notably, women make up only one-third of authors and are underrepresented in senior author positions in MM studies [68].

#### 5.2.2. Clinical Manifestations

The clinical picture shows obvious sex differences in the genetic and molecular profile. Females show significantly higher rates of moderate and severe anemia (*p* < 0.001) and over-representation of cytogenetic abnormalities compared to males [69]. Although both sexes share common symptoms, such as bone pain, anemia, and renal failure [69], women are more likely to have cytogenetic abnormalities and IGH translocations, and men have hyperdiploidy. Specific numerical (+1 and −13) and structural abnormalities (1p and 17p modifications) detected by cytogenetics showed over-representation in females compared to males [66]. In a retrospective registry study of 1312 patients (476 male to male (M→M); 334 female to male (F→M); 258 male to female (M→F); and 244 female to female (F→F)) reported to the European Group for Blood and Marrow Transplantation (EBMT), the median age at diagnosis was 62 years (363 men with a median age at diagnosis 62 years and 292 women with a median age at diagnosis 63 years, *p* = 0.086) [70]. In a unicentric retrospective analysis of patients with MM treated at a tertiary cancer center between 2003 and 2018 involving 655 patients (median age at diagnosis 62 years; 363 men with a median age at diagnosis 62 years and 292 women with a median age at diagnosis 63 years, *p* = 0.086), ref. [67] researchers found that most patients (*n* = 561, 86%) received myeloma-specific treatment. They also reported that women were more likely to have moderate and severe anemia (*p* < 0.001). Serum protein electrophoresis consistently shows the presence of M bands in both sexes, although interpretation may need to consider sex-specific differences in disease biology [69].

#### 5.2.3. Impact on Prognosis

The relationship between sex and MM survival is complex. Some studies have reported improved overall survival in women [6], with one study demonstrating a significant overall survival benefit for females after controlling for clinical factors [6], whereas others have found no significant differences, especially in autologous stem cell transplantation [71]. Sex differences in prognostic factors include higher rates of comorbidity at diagnosis in men, higher incidence of high-risk cytogenetic abnormalities and immunoglobulin heavy chain gene translocations in women, and higher prevalence of hyperdiploidy in men. FISH studies indicated that +1q and +7 were noticeably higher in females [66,67]. In a study by Derman et al. [6], OS and PFS were significantly better for females than males. The OS benefit persisted for females (SEER: HR = 0.92, 95% CI 0.90–0.94, *p* < 0.0001; MMRF: HR = 0.66, 95% CI 0.51–0.85, *p* < 0.0001) but was attenuated for PFS (MMRF: HR = 0.85, 95% CI 0.72–1.00, *p* = 0.06) after controlling for age, International Staging System, performance status and autologous stem cell transplant. Conflicting evidence on sex differences in MM outcomes [65] highlights the need for larger, more comprehensive studies.

#### 5.2.4. Treatment Considerations

Although overall survival is similar between men and women [67,71], population-based studies suggest that women may have a survival advantage [6]. The best overall survival (OS) from the time of transplantation was found in F→F (median 41 months), with no significant difference between other groups (median 25 months in M→M, 18 months in F→M, and 19 months in M→F) despite significantly higher non-relapse mortality in M→F. However, this was due to a significantly lower relapse rate (REL) in F→M compared to all other groups [70]. Toxicities after ASCT were not significantly different between the sexes, with the exception of severe mucositis, which occurred in 22% of men versus 40% of women (*p* = 0.001), and higher exposure to treosulfan is associated with higher mortality [67,72]. In a retrospective analysis of 112 multiple myeloma (MM) patients treated with first-line high-dose chemotherapy (HDCT) with treosulfan and melphalan (TreoMel) followed by ASCT at a single academic center between January 2020 and August 2022, researchers found significant sex-specific differences in treosulfan exposure. Females had higher peak levels (343.8 vs. 309.0 mg/L, *p* = 0.0011) and area under the curve (AUC) (869.9 vs. 830.5 mg*h/L, *p* = 0.0427) compared to males. Higher treosulfan exposure was associated with increased mortality in females but not in males. Females with treosulfan AUC > 900 mg*h/L had significantly shorter overall survival, while PFS was unaffected by treosulfan exposure [72]. Treatment decisions also vary, with women placing more importance on emotional state and quality of life. A survey of 289 RRMM patients (mean age 66 ± 9 years, 53% female) revealed significant sex-specific differences in treatment decision making. Women rated potential side-effect severity as very or extremely influential more often than men (51% vs. 38%), with mean scores of 3.4 ± 1.3 for women versus 3.2 ± 1.1 for men (*p* < 0.05). Supportive care requirements were also more influential for women (2.5 ± 1.4 vs. 2.1 ± 1.3, *p* < 0.05). Regarding quality of life impact, 61% of women rated it very or extremely influential compared to 49% of men (*p* = 0.018), with higher mean scores for women (3.6 ± 1.3 vs. 3.2 ± 1.3, *p* < 0.05). The emotional state was rated higher by women at first therapy change (2.6 ± 1.4 vs. 2.0 ± 1.2, *p* < 0.05), with this gap widening for subsequent therapy changes (2.7 ± 1.4 vs. 1.8 ± 1.2, *p* < 0.05). Physical condition (frailty) had more influence on women’s decisions (2.8 ± 1.4 vs. 2.2 ± 1.3, *p* < 0.05), with more women rating it very or extremely influential (37% vs. 21%, *p* = 0.03). Both sexes primarily relied on medical team recommendations (F: 89%; M: 92%, almost or every time), followed by patient advocacy myeloma websites, which women used more frequently (F: 3.8 ± 1.2 vs. M: 3.5 ± 1.2, *p* < 0.05) and increasingly over subsequent relapses (F: 4.2 ± 0.9 vs. M: 3.6 ± 1.2, *p* < 0.05) [73]. Cytogenetic and FISH abnormalities are more frequent in women, which may affect response to treatment [66]. This highlights the need for sex-specific considerations in treatment strategies from dosage adjustments to supportive care.

### 5.3. Clinical Implications

#### 5.3.1. Common Patterns

The study of sex differences in hematopoietic malignancies displays remarkable patterns that go beyond just the presence of disease. Although men are more likely to develop both leukemia and multiple myeloma, women often have better odds of survival despite experiencing more severe treatment-related toxicity. Women with CML exhibit a remarkable pattern, as treatment-free remissions are frequently observed, and they have faster molecular responses.

These diseases have molecular signatures that exhibit distinct sex-specific variations. The frequency of AML mutations in women is higher than that in men, with FLT3-ITD, NPM1, and DNMT3A mutations occurring more frequently. The presence of IGH translocations and del(13q) is a common feature in women with multiple myeloma, while men exhibit hyperdiploidy. These consistent molecular differences point to fundamental biological mechanisms underlying sex-specific disease expression.

In these malignancies, treatment responses reveal a fascinating treatment paradox: women have more severe treatment-related toxicity but overall better outcomes. In CML, women exhibit a stronger response to TKI therapy (higher MMR rates (80% vs. 45%, *p* = 0.018)) [32,33]. Despite higher intolerance rates, ALL females with more severe toxicity show better survival rates [58].

#### 5.3.2. Clinical Applications

These consistent patterns have significant implications for clinical practice. Sex-specific dosing strategies should be considered when modifying treatment, especially for agents known to have different toxicity profiles. For example, the variability in anthracycline cardiac toxicity at different stages of a woman’s life (higher risk in prepubertal females and lower risk in premenopausal females) suggests the need for age- and sex-specific dosing protocols.

Sex-specific risk patterns should be taken into consideration when developing further surveillance suggestions. It will be crucial to better monitor cardiac toxicity in women using anthracycline, particularly when treating AML. The necessity for focused neurological surveillance is indicated by the various forms of cognitive damage seen in female ALL survivors. Because cytogenetic abnormalities are more common in women with multiple myeloma, more rigorous molecular surveillance is necessary.

**Figure 2:**
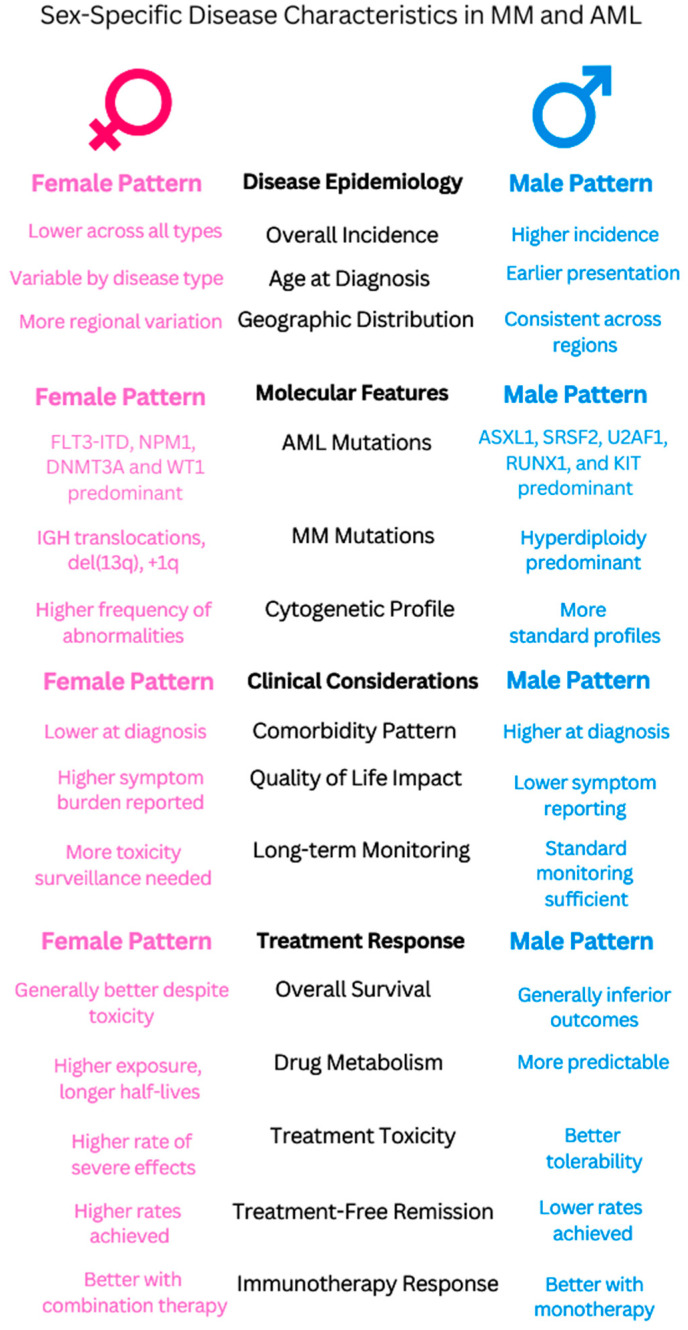
Comparative analysis of sex-specific characteristics in multiple myeloma (MM) and acute myeloid leukemia (AML). The figure illustrates key differences between female (pink, **left**) and male (blue, **right**) patterns across four major categories: disease epidemiology, molecular features, clinical considerations, and treatment response.

There are several crucial avenues for the future advancement of sex-specific strategies in hematological cancers. To overcome the existing gap in oncology studies that publish sex-specific results, sex-specific analysis should be consistently included in clinical trial design. The molecular underpinnings behind various treatment responses, particularly the part played by immune system variations and hormonal variables, require targeted research. Patient outcomes could be enhanced by creating standardized toxicity management procedures and sex-specific risk assessment instruments (Figure 2).

## 6. Gender and Hematologic Malignancies: Sociocultural and Clinical Implications

*Treatment Decisions and Adherence*: Research has found significant sex-specific disparities in hematopoietic malignancies and their treatment. Women receive less funding from the NIH for research in this field [74] and experience poorer physical health and greater pain and symptom burden compared to men [75]. Gender differences have been found in medication adherence and guideline-based care, with women being less adherent and less likely to receive recommended care [76]. These differences also extend to survivors’ quality of life and symptoms [75]. Racial and ethnic disparities exist in incidence, survival, and outcomes [77]. Factors contributing to these inequalities include genetic ancestry, socioeconomic status, and geographic region [78]. Increased awareness of sex differences in disease risk and outcomes is needed among clinicians [38] as well as comprehensive strategies to reduce inequities in the healthcare system.

*Psychosocial Support and Survivorship*: Studies investigating psychosocial support and survivorship among individuals with hematologic malignancies suggest there might be gender disparities. Women report higher levels of distress and seek more help compared to men [79]. They also showed worse physical health, more pain, and a higher symptom burden than males in the analysis of Tinsley-Vance et al. (2023). Female gender was associated with higher vulnerability to symptoms of PTSD [75], as found by Liu et al. in 2015. The most common unmet needs of patients include psychological, informational, and family-related problems, while fear of recurrence is also widely reported [80,81]. Although attempts have been made to eliminate gender bias in cancer research, concerns about the representation and study of women and men in the medical and psychosocial literature persist [82].

## 7. Translating Biology to Clinical Practice: The Future of Precision Medicine

The broad-based evidence of sex and gender differences in hematologic malignancies creates an apparent need for translating biological findings into clinical applications. This could help advance precision medicine approaches that optimize patient outcomes.

### 7.1. Integrating Sex and Gender into Clinical Trials

The current literature evidences significant gaps in sex-specific reporting. This deficiency should be addressed by the systematic inclusion of both sex and gender variables in clinical trials, using standardized reporting protocols that capture sex-specific molecular profiles, treatment responses, and toxicity patterns. The clear sex-specific trends in treatment outcomes—including the paradoxical better survival of females with ALL despite increased toxicity and superior responses to immune checkpoint inhibitor monotherapy among males as reported by Wang et al. in 2019 [83]—represent key evidence of the need for sex-stratified analysis in clinical studies.

### 7.2. Development of Sex- and Gender-Sensitive Clinical Guidelines

Evidence for sex-specific treatment responses across various hematologic malignancies necessitates clinical guidelines regarding specific sexes. For instance, the reported divergent responses to TKI therapy in CML (where females achieve 80% MMR rates compared to 45% in males (*p* = 0.018) despite higher intolerance rates) shows that females achieve major molecular response quicker but with a higher intolerance rate to the treatment [57], requiring a sex-specific approach in treatment modalities. This should be guided by dosing strategies, monitoring protocols, and toxicity management.

The distinct molecular profiles between sexes, such as higher frequencies of FLT3-ITD, NPM1, and DNMT3A mutations in female AML patients [49], form a basis for developing targeted therapies. Understanding these biological mechanisms can help develop treatment strategies in a sex-specific manner that considers differences in drug metabolism, immune responses, and molecular characteristics. This is in tandem with the broader goals of precision medicine: treatment optimization based on the peculiarities of individual patients.

### 7.3. Eliminating Gender Disparity in Healthcare

In research involving multiple myeloma, marked inequalities between the genders exist, with women constituting only one-third of authors and being underrepresented in senior authorship positions. Recent studies have highlighted significant gender disparities in multiple myeloma research authorship. Women constitute only about one-third of authors in multiple myeloma publications, with even lower representation in senior authorship roles [68]. Similar disparities are observed in CAR-T clinical trials for lymphoma and myeloma, where female authorship is around 29% [84]. These disparities extend beyond myeloma to other areas of medicine, including neurology and general medical publications [85]. Interestingly, gender differences have been noted in the prevalence of genetic lesions in multiple myeloma patients, which may impact clinical outcomes [86]. The way gender-specific issues are researched and addressed in the field is likely influenced by this underrepresentation. While the reasons for these disparities are not fully understood, factors such as underrepresentation in academic positions, unconscious bias, and historical inequalities likely contribute [87]. These disparities may stem from structural barriers in academic career progression, including limited access to mentorship, lower grant funding success rates, and work–life balance challenges that disproportionately affect women in research. Additionally, publication and peer review processes may contain implicit biases that disadvantage female researchers, further perpetuating the gender gap in authorship. Recent trends suggest some improvement in gender equality, particularly in pediatric studies and publications since 2021 [84].

To tackle these systemic disparities, a multifaceted strategy is essential, encompassing several key approaches: increasing the inclusion of women in research leadership positions to ensure diverse perspectives in study design and interpretation; implementing gender-sensitive monitoring and research practices to promote a more nuanced understanding of gender-specific factors in hematologic malignancies; creating focused initiatives to remove gender-specific obstacles in patient care and academic medicine, addressing barriers that women face in both receiving care and advancing in their careers; and developing supporting initiatives that consider the psychosocial needs of patients and gender-specific obstacles in medical professions, which can lead to more effective and equitable healthcare systems. Academic institutions could establish targeted mentorship programs, transparent promotion criteria, and family-friendly policies, while journals and funding agencies might implement blind review processes and balanced evaluation committees. Professional societies in hematology could further support these efforts by creating dedicated platforms to highlight women’s research contributions and establishing awards that recognize female excellence in multiple myeloma research. The medical community can strive towards more equitable and effective treatment strategies that optimize outcomes for all patients with hematologic malignancies by systematically addressing these issues in both patient care and the medical profession.

## 8. Conclusions

This review highlights how sex and gender bear heavily on the biology, presentation, and treatment outcomes of hematologic malignancies, specifically MM and AML. Males uniformly have increased disease incidence and often poorer outcomes in many malignancies, though females commonly show superior survival with increased treatment toxicities. The underlying sex-specific molecular profiles, immune responses, and patterns of drug metabolism support the need for tailored approaches.

Despite these obvious patterns, significant knowledge gaps persist. Further research is required to explain the mechanisms behind sex-based differences in disease biology and treatment responses. The influence of gender-related sociocultural factors on access to healthcare, adherence to treatment, and outcomes must also be duly investigated.

Integration of sex and gender issues in precision medicine is a key step toward the best possible care for the patient. The development of sex-specific treatment protocols, monitoring strategies, and clinical guidelines will move closer to truly personalized medicine, ensuring better outcomes in all patients with hematologic malignancies.

## Data Availability

The data supporting this study’s findings are included within the article and are available upon request from the corresponding author.

## Abbreviations

The following abbreviations are used in this manuscript:

AML	Acute Myeloid Leukemia
ALL	Acute Lymphoblastic Leukemia
CML	Chronic Myeloid Leukemia
MM	Multiple Myeloma
B-ALL	B-cell Acute Lymphoblastic Leukemia
T-ALL	T-cell Acute Lymphoblastic Leukemia
BCR-ABL	Breakpoint Cluster Region-Abelson
FLT3-ITD	FMS-like Tyrosine Kinase 3 Internal Tandem Duplication
DNMT3A	DNA Methyltransferase 3 Alpha
NPM1	Nucleophosmin 1
WT1	Wilms Tumor 1
ASXL1	Additional Sex Combs Like 1
SRSF2	Serine/Arginine-Rich Splicing Factor 2
U2AF1	U2 Small Nuclear RNA Auxiliary Factor 1
RUNX1	Runt Related Transcription Factor 1
IGH	Immunoglobulin Heavy Chain
del(13q)	Deletion of chromosome 13q
+1q	Addition of chromosome 1q
SNPs	Single Nucleotide Polymorphisms
TKI	Tyrosine Kinase Inhibitor
CNS	Central Nervous System
WBC	White Blood Cell
FISH	Fluorescence In Situ Hybridization
NIH	National Institutes of Health
PTSD	Post-Traumatic Stress Disorder
BSA	Body Surface Area
ESMO	European Society for Medical Oncology
COG	Children’s Oncology Group
AMLCG	AML Cooperative Group
SLA	Service Level Agreement
DNA	Deoxyribonucleic Acid
RNA	Ribonucleic Acid
OS	Overall Survival
PFS	Progression-Free Survival
MMR	Major Molecular Response
CCyR	Complete Cytogenetic Response
SEER	Surveillance, Epidemiology, and End Results
MMRF	Multiple Myeloma Research Foundation
ASCT	Autologous Stem Cell Transplantation
EBMT	European Group for Blood and Marrow Transplantation
HDCT	High-Dose Chemotherapy
TreoMel	Treosulfan and Melphalan
AUC	Area Under the Curve
RRMM	Relapsed/Refractory Multiple Myeloma
EFS	Event-Free Survival
EAPC	Estimated Annual Percentage Change
LOY	Loss of Y Chromosome
HSPCs	Hematopoietic Stem and Progenitor Cells
A:N	Abnormal-to-Normal (ratio)
CI	Confidence Interval
HR	Hazard Ratio
REL	Relapse Rate
